# Biophysical and Biological Tools to Better Characterize the Stability, Safety and Efficacy of a Cosmeceutical for Acne-Prone Skin

**DOI:** 10.3390/molecules27041255

**Published:** 2022-02-13

**Authors:** Sabrina Sommatis, Maria Chiara Capillo, Cristina Maccario, Elsa Liga, Giulia Grimaldi, Raffaele Rauso, Pier Luca Bencini, Stefania Guida, Nicola Zerbinati, Roberto Mocchi

**Affiliations:** 1 UB-CARE S.r.l.-Spin-Off University of Pavia, 27100 Pavia, Italy; sabrina.sommatis@ub-careitaly.it (S.S.); mariachiara.capillo@ub-careitaly.it (M.C.C.); cristina.maccario@ub-careitaly.it (C.M.); quality@ub-careitaly.it (E.L.); research@ub-careitaly.it (G.G.); 2 Maxillofacial Surgery Unit, Department of Medicine and Surgery, University of Campania “Luigi Vanvitelli”, 80138 Naples, Italy; raffaele.rauso@unicampania.it; 3 Istituto di Chirurgia e Laser-Chirurgia in Dermatologia (I.C.L.I.D.), 20121 Milan, Italy; pl.bencini@iclid.it; 4 Dermatology Unit, Department of Surgical, Medical, Dental and Morphological Sciences Related to Transplant, Oncology and Regenerative Medicine, University of Modena and Reggio Emilia, 41124 Modena, Italy; stefania.guida@unimore.it; 5 Department of Medicine and Surgery, University of Insubria, 21100 Varese, Italy

**Keywords:** acne-prone skin, *Cutibacterium acnes*, rheological properties, keratinocytes, human reconstructed 3D models, biofilm, antimicrobial effectiveness, anti-inflammatory activity, preservative system, cosmeceutical

## Abstract

(1) Background: Acne is a widespread skin disease, especially among adolescents. Following the COVID-19 pandemic and the use of masks, the problem has been affecting a greater number of people, and the attention of the skin care beauty routine cosmetics has been focused on the “Maskne”, caused by the sebum excretion rate (SER) that stimulates microbial proliferation. (2) Methods: the present study was focused on the rheological characterization and quality assurance of the preservative system of an anti-acne serum. The biological effectiveness (cytotoxicity—skin and eye irritation—antimicrobial, biofilm eradication and anti-inflammatory activity) was evaluated in a monolayer cell line of keratinocytes (HaCaT) and on 3D models (reconstructed human epidermis, RHE and human reconstructed corneal epithelium, HCE). The *Cutibacterium acnes*, as the most relevant acne-inducing bacterium, is chosen as a pro-inflammatory stimulus and to evaluate the antimicrobial activity of the serum. (3) Results and Conclusions: Rheology allows to simulate serum behavior at rest, extrusion and application, so the serum could be defined as having a solid-like behavior and being pseudoplastic. The preservative system is in compliance with the criteria of the reference standard. Biological effectiveness evaluation shows non-cytotoxic and irritant behavior with a good antimicrobial and anti-inflammatory activity of the formulation, supporting the effectiveness of the serum for acne-prone skin treatment.

## 1. Introduction

Acne is a chronic pilosebaceous inflammatory disease most often, but not only, seen in adolescents. The pathogenesis is multifactorial and includes genetics, hormones, lifestyle and several bacterial species, factors that combined play a key role during the onset and progression of the disorder. The main biological events are androgen stimulation and hyper-seborrhea, follicular hyper-keratinization and desquamation of infundibular epithelium, colonization and proliferation of *Cutibacterium acnes* (*C. acnes*) with stimulation of a local innate immune response [1,2]. Microbiologically, the outer surface of adult skin is colonized by resident microflora, normal flora or indigenous microbiota that represents an integral part of the innate immune system. The density and composition of the skin’s indigenous microflora varies with anatomical site, people age, sex, level of hygiene and type of cleansers used, but also extrinsic factors such as the climate, temperature and humidity, which can significantly affect microbial numbers and composition [3]. Commonly, micrococci with coagulase-negative staphylococci, *Peptococcus* spp., *Micrococcus* spp., diphtheroids with corynebacteria and *Brevibacterium* spp., propionibacteria and Gram-negative rods are the main constituents of the skin-resident flora. However, the indigenous microbiota of the skin is also considered a potential source of infection, particularly when there is a skin injury or a disruption to the skin’s normal microbiological balance [4]. The Gram-positive, anaerobic, aerotolerant, bacillus-shaped *C. acnes* bacterium is a major resident of the human skin microbiota but also the most frequent cause of opportunistic infection with an optimal growth according to skin environmental conditions, temperature ranging from 31 to 37 °C and pH ranging between 4.2 and 7.9 [5,6]. Recently, the *C. acnes* ability to form biofilm in pilosebaceous unit (PU) was confirmed and identified as a major factor in the pathogenesis of the acne vulgaris [7,8]. Microbial over-colonization in the SG and biofilm organization are involved in the inflammatory cascade interacting with Toll-like receptor (TLR) and stimulating androgen secretion. SGs are localized at multiple sites in normal skin where they express several different cytokines at steady state as interleukin (IL)-1α, IL-1β and tumor necrosis factor (TNF)-α [9]. The presence of *C. acnes* bacteria promotes the formation of propionic and acetic acid, the release of extracellular enzymatic products such as proteases, lipases, and hyaluronidases inducing irritation, inflammation and efflux of inflammatory mediators as IL-1α and IL-8 in dermis and surrounding tissues via TLR2 [10,11].

Currently, high temperature, sweat and increased humidity have been studied as risk factors associated with “Maskne”, an acne flare due to long-term mask wearing during the COVID-19 pandemic. The lesions are mainly found in the area covered by the mask, and recent studies classified different forms ranging from moderate to severe [12,13]. The humid environment and the rise in temperature caused by expired air and perspiration affect the sebum excretion rate (SER), resulting in a defective barrier function that facilitates the exchange of materials between the follicle and surrounding tissue that stimulates microbial proliferation [10,12,14]. Human sebum consists mainly of squalene, triglycerides, wax esters and a small amount of cholesterol and derivatives. The SER, as well as the squalene concentration in the skin, is directly affected by temperature, with the sebum excretion increasing by approximately 10% for each 1 °C rise [10]. Moreover, changes in both the surface sebum composition and skin moisturizing could contribute to skin barrier alterations, leading to an influx of water and nutrients into the follicle that promotes bacterial microflora dysbiosis [10,12]. The use of the mask today remains one of the main approaches to protect ourselves and others, but taking oculate precautions, such as frequently changing masks or washing the face with appropriate cleanser, is highly recommended to reduce some unavoidable negative effects related to prolonged use. In particular, for people with acne-prone skin, the application of topical formulation containing emollients and oil-control ingredients is strongly suggested to alleviate the undesirable effects and maintain skin compliance [15]. 

The biological mechanism of acne onset and progression should be best understood in order to design functional formulations for the effective treatment of the disorder. Hence, topical formulations for treating acne-prone skin should contain keratolytic, anti-seborrheic, antimicrobial and anti-inflammatory active ingredients [1]. In order to be effective, formulations must have physical and chemical properties that allow a correct absorption across the skin and a control of functional qualities for the end-user satisfaction. The application of a cosmetic product to the surface of the human skin can take place with different motions, stroke and shear rate: knowledge of the rheological properties of a cosmetic is an essential key to reproduce its use, skin feel and spreadability [16]. Formulation, preparations, material packaging and shelf storage are associated with a complex flow of materials. Products flow both when they are pumped through a dispenser and when they are smeared with the hands, but also within the packaging when they are subjected to external stimuli. Since the application and acceptance of a healthcare product are also dependent by flow properties of the final product, rheological measurements are necessary to make pharmaceuticals and cosmetics optimal for their distinctive needs [17]. The present study has been focused on a full quality-control assessment based on the biophysical behavior but also the safety and efficacy profile of a serum for acne-prone skin. A rheological study allows to define if during the storage the product’s ingredients should not separate from each other in the container. Moreover, the study evaluates if, when applied on the human skin, the product spreads without feeling greasy or sticky and if the cosmetic is too hard or too runny when it is poured or squeezed from the packaging [18]. Once the biophysical features of the product have been characterized, the experimental design was focused on the investigation about the microbial stability of the preservative system and the in vitro antimicrobial and anti-inflammatory activity against the most relevant acne-inducing bacterium (*C. acnes*) on keratinocytes monolayer cell culture and 3D skin models (reconstructed human epidermis, RHE). The potential skin and eye irritation risk was also investigated according to standard references on RHE and human reconstructed corneal epithelium (HCE) models.

## 2. Results

### 2.1. Rheological Characterization

The shear viscosity of the tested serum decreases with an increase in the shear rate, demonstrating the product’s non-Newtonian shear thinning behaviour (Pseudoplastic). As shown in Table 1, at a low shear rate, the serum has high viscosity caused by the network structure promoted by the carbomer. The increase in the shear rate influences the viscosity of the serum, which decreases as a consequence of the destruction and deformation of its internal structure. The shear thinning behaviour becomes essential for a serum during its spreading because the cosmetic should be evenly spread on the skin in order to guarantee a homogeneous and complete absorption of the product [18]. 

**Figure 1:**
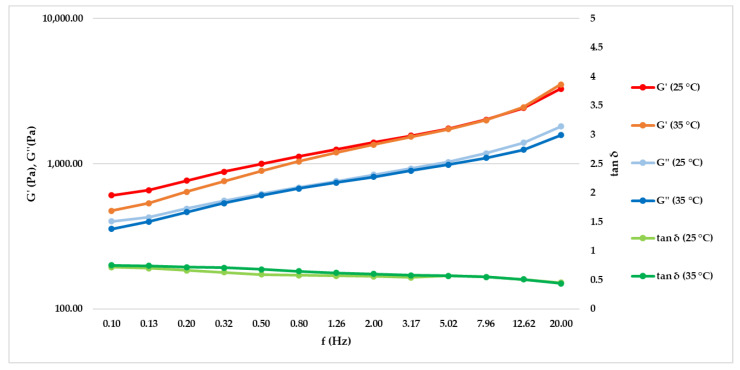
The graph shows the G’, G’’ and tan δ behaviors at 25 and 35 °C varying the frequency between 0.1 and 20 Hz at a fixed shear strain (*n* = 1, replicates = 3).

A preliminary amplitude sweep test is performed and 0.2% is identified in the linear viscoelastic region (LVER) as the shear strain value at which it is possible to make a deformation without breaking the sample. The frequency sweep test is then performed; in Figure 1, it is possible to notice that the storage modulus (G’) and loss modulus (G’’) are increased as a result of the frequency value increase. The storage modulus is always greater than the loss modulus within the studied frequency range. Therefore, the elastic nature of the serum prevails over the viscous nature. From the ratio between G’ and G’’, the tangent of the phase angle (tan δ) value lower than unity is obtained, exhibiting a solid-like behaviour.

### 2.2. Evaluation of the Antimicrobial Stability of the Preservative System

Data collected confirmed the assurance of the formulation preservative system in a period of 28 days. The log reduction at each time point analyzed the results in compliance with the criteria imposed from the reference standard method [20]. In particular, as shown in Table 2, after 7 days (t7), a reduction of at least three logs for bacteria *Pseudomonas aeruginosa* (*P. aeruginosa* ATCC 9027) *Staphylococcus aureus* (*S. aureus* ATCC 6538) and *Escherichia coli* (*E. coli* ATCC 87394) and one log for *Candida albicans* (*C. albicans* ATCC 10231) was observed; after 14 days (t14), a reduction of at least three logs for bacteria (without any increase compared to the previous time), at least one log for *C. albicans* (without any increase with respect to the previous time), and no increase compared to starter inoculum for *Aspergillus brasiliensis* (*A. brasiliensis* ATCC 16404) were registered; after 28 days (t28), a reduction of at least three logs for bacteria (without any increase compared to t14), at least one log for *C. albicans* (without any increase compared to t14), and at least one log for *A. brasiliensis* were observed.

### 2.3. Antimicrobial Activity against C. acnes

**Figure 2:**
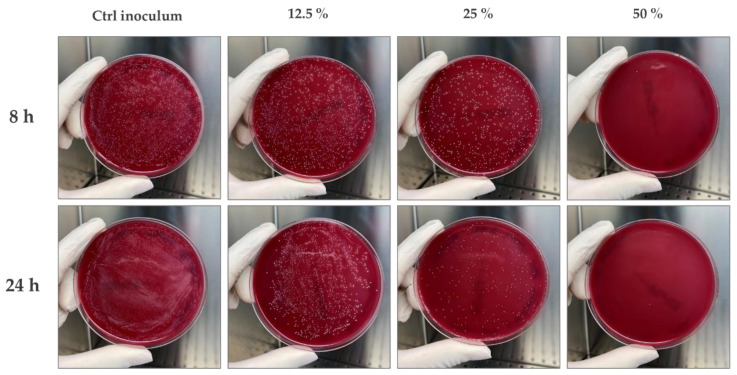
Representative images of the sheep blood agar plates after different incubation times (8–24 h) between the *C. acnes* strain (10^4^ CFU/mL final concentration) and different concentrations of the serum (12.5%–25%–50%) diluted in BHI. Ctrl inoculum: *C. acnes* in BHI at 10^4^ CFU/mL final concentration (*n* = 2, replicates = 3).

The obtained results show that the anti-acne serum determines a visible dose-dependent growth inhibition of the *C. acnes* strain already after 8 h of contact, starting from the concentration of 50%. Figure 2 shows, for each contact time analysed, the first concentration able to reduce the microbial load (50% with a reduction ≥ 99.9%), the concentration that shows an inhibition in microbial load (25%) and the lower concentration (12.5%) with growth comparable to control (*Ctrl inoculum*). 

### 2.4. Evaluation of Biofilm Eradication Activity

**Figure 3:**
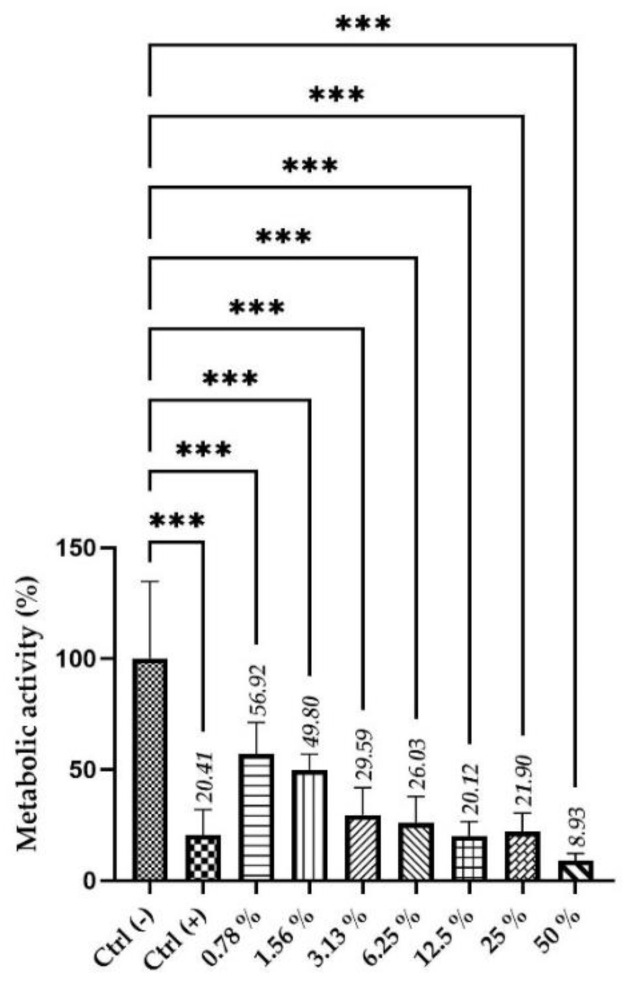
Biofilm metabolic activity (%) of a preformed biofilm after 24 h of treatment with BHI as Ctrl (-), Resveratrol (394 µg/mL) as Ctrl (+) and different concentrations of the serum (0.78–50%). (*n* = 2, replicates = 3). The explanation of *** is in the paragraph 4.12. (Statistical analysis).

To determine the metabolic activity of microbial cells inhabiting the biofilm, a colorimetric method based on the reduction of a tetrazolium salt was performed [21,22]. The serum at different concentrations (0.78–50%) applied over the preformed biofilm shows a significative reduction in the biofilm metabolic activity in a concentration-dependent manner, as shown in Figure 3. Resveratrol, reported in the literature as a candidate for *C. acnes* biofilm eradication, shows a significative percentage of eradication equal to 80% at 394 µg/mL concentration.

### 2.5. Cytotoxicity/Viability Assay on HaCaT Cells

**Figure 4:**
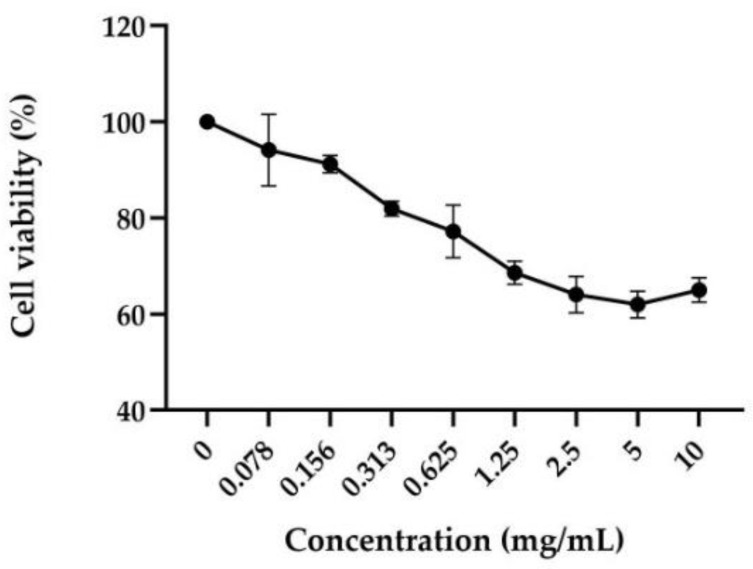
Cell viability of HaCaT cells (% ± SD) after treatment with anti-acne serum at different concentrations (0.078–10 mg/mL) for 8 h. Cell viability was assessed through MTT assay (*n* = 3, replicates = 3).

The anti-acne serum has shown high biocompatibility in human skin keratinocytes. The cytotoxicity test was required to evaluate the effect of the product on cellular viability and to select the appropriate concentrations that did not cause a decrease in cell respiration exceeding 20% for the following anti-inflammatory test. In Figure 4, the results of the HaCaT viability after treatment (8 h) with different concentrations of the tested product (range 0.078–10 mg/mL) are shown, expressed as a percentage compared to the control (untreated cells). The serum concentrations 0.078–0.156–0.313 mg/mL were selected for the following assay since they showed a cell viability higher than 80%.

**Figure 5:**
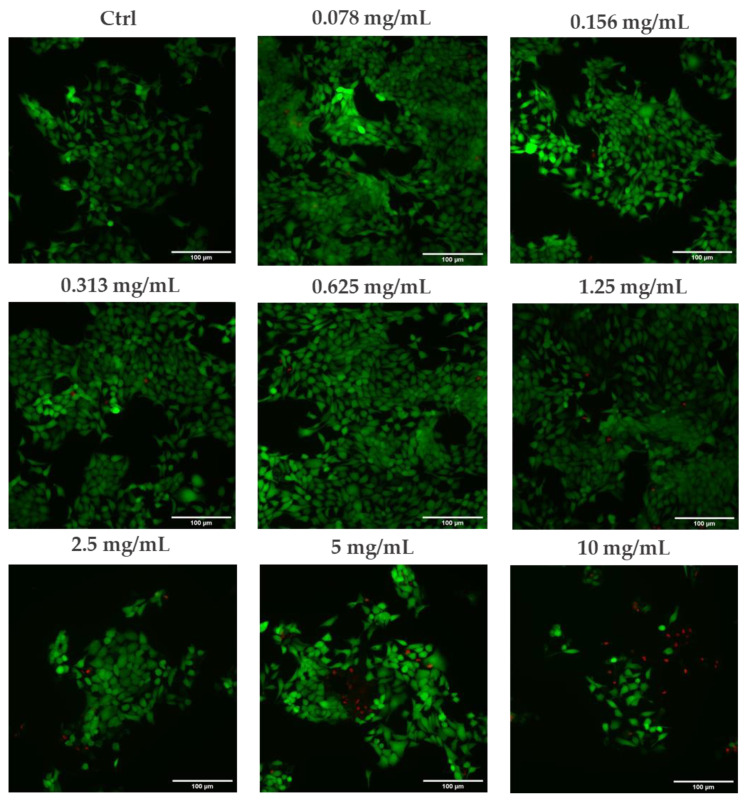
Fluorescence microscopic images of in vitro live (Calcein AM in green) and dead (EthD-1 in red) staining of HaCaT cells after 8 h of treatment with different serum concentrations (0.078–10 mg/mL) at 20× magnification (Scale bar: 100 μm) (*n* = 1; replicates = 2).

Data obtained with the MTT test were confirmed by the LIVE/DEAD^®^ Viability/Cytotoxicity staining of the HaCaT cells treated with the same serum concentrations (0.078–10 mg/mL), as shown in Figure 5. The green (Calcein Acetoxymethyl, Calcein AM) and red (Ethidium homodimer-1, EthD-1) fluorescence staining provided by the kit indicated live and dead cells, respectively. Fluorescence microscopic images obtained with a DM6B Leica Widefield (Wetzlar, Germany) microscope confirmed an increase in the number of dead cells at the higher serum concentrations tested compared to untreated cells (Ctrl).

### 2.6. Anti-Inflammatory Activity of the Anti-Acne Serum on HaCaT Cells

**Figure 6:**
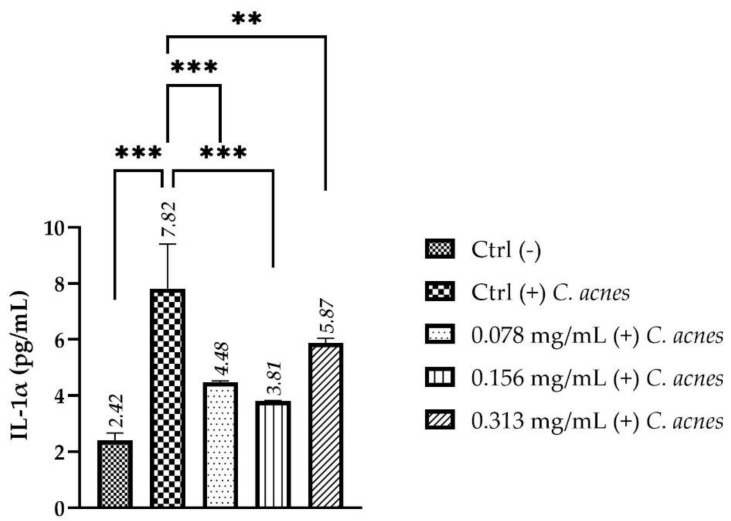
IL-1α levels after *C. acnes* stimulation on HaCaT cells. Values are expressed as concentration in pg/mL; Ctrl (-): untreated cells; Ctrl (+) *C. acnes*: untreated cells stimulated with *C. acnes*; 0.078–0.156–0.313 mg/mL (+) *C. acnes*: cells treated with different concentrations of acne stress control serum stimulated with *C. acnes* (*n* = 2, replicates = 2). The explanation of ** and *** is in the paragraph 4.12. (Statistical analysis).

The possible activity on inflammatory cytokines of the tested serum after stimulation with *C. acnes* was evaluated on HaCaT cells, measuring the expression of IL-1α by an enzyme-linked immunosorbent assay (ELISA) technique. The IL-1α levels were expressed as the concentration in pg/mL in HaCaT cells treated with the functional formulation at the concentrations of 0.078–0.156–0.313 mg/mL compared to positive control (Ctrl+, cells stimulated only with *C. acnes*), as shown in Figure 6. The acne stress control serum shows an evident effect on the release of the IL-1α, following a pro-inflammatory stimulus (*C. acnes*), leading to a statistically significant reduction in the cytokine levels after 8 h of treatment, at all tested concentrations compared to stimulated control (Ctrl + *C. acnes*).

### 2.7. Evaluation of Skin Irritation on Reconstructed Human Epidermis (RHE)

**Figure 7:**
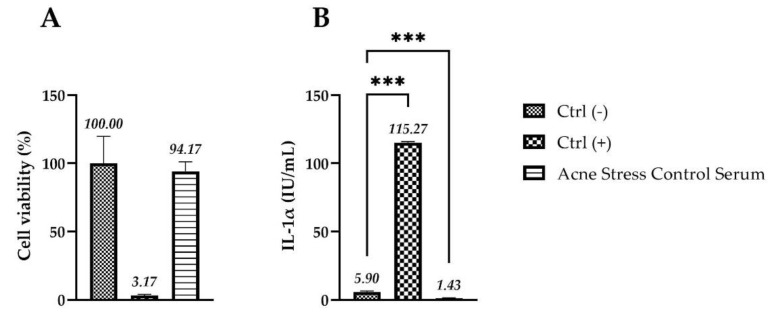
(**A**) Cell viability expressed as a percentage (%) compared to negative control Ctrl (-): RHE treated with DPBS; Ctrl (+): RHE treated with SDS; acne stress control serum: RHE inserts treated for 42 min with the serum. (**B**) IL-1α amount (IU/mL) in medium after treatment with acne stress control serum; Ctrl (-): RHE treated with DPBS; Ctrl (+): RHE treated with SDS; acne stress control serum: RHE inserts treated for 42 min with the serum (*n* = 1, replicates = 4). The explanation of *** is in the paragraph 4.12. (Statistical analysis).

Skin irritation was investigated on a 3D RHE model, evaluating cell viability by MTT assay and the IL-1α amount by an ELISA kit. As reported in Figure 7, the 3D model RHE treated with the serum shows a viability greater than 50% and an amount of interleukin IL-1α released lower than 9 international units (IU)/mL (thresholds used to identify a substance as not irritant, according to ISO 10993-10: 2010 Guideline) [23].

### 2.8. Evaluation of Eye Irritation on Human Reconstructed Corneal Epithelium (HCE)

**Figure 8:**
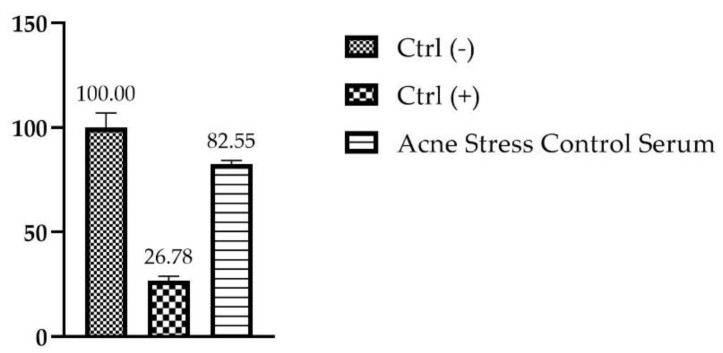
Cell viability expressed as a percentage (%) compared to negative control Ctrl (-): RHE treated with DPBS following treatment for 30 min of the RHE inserts with the serum; Ctrl (+): RHE treated with methyl acetate (*n* = 1, replicates = 3).

Eye irritation was investigated on 3D HCE, evaluating cell viability by MTT assay according to the standard method [24]. As reported in Figure 8, the HCE model treated with the serum shows a viability greater than 60% (threshold used to identify a substance as not dangerous or irritating for eye), making it safe to use in the periocular zone.

### 2.9. Anti-Inflammatory Activity on RHE Model C. acnes Stimulated

**Figure 9:**
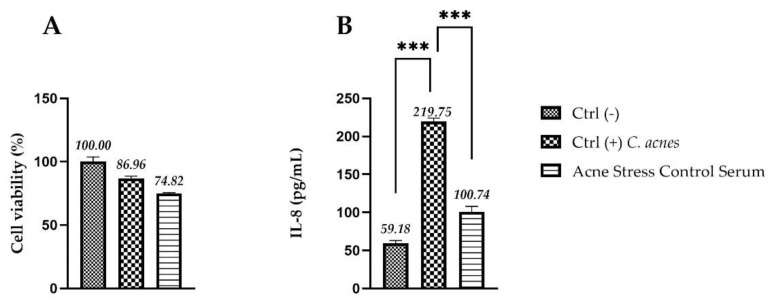
(**A**) Cell viability expressed as a percentage (%) compared to negative control Ctrl (-): RHE treated with DPBS; acne stress control serum: RHE inserts stimulated with *C. acnes* and treated for 24 h with the serum; Ctrl (+): RHE stimulated with *C. acnes* and treated with DPBS. (**B**) IL-8 amount (pg/mL) in medium after treatment with acne stress control serum in RHE inserts infected with *C. acnes*; Ctrl (-): cells treated with DPBS; Ctrl (+): RHE stimulated with *C. acnes* and treated with DPBS (*n* = 1, replicates = 3). The explanation of *** is in the paragraph 4.12. (Statistical analysis).

The soothing effect of the serum was evaluated quantifying the expression of IL-8 by an ELISA assay after media stimulation with *C. acnes* in a RHE model. The viability was performed in order to verify that the experimental model adopted remains in compliance with the acceptability criteria of the standard method (viability ≥ 50%) [23]. The IL-8 levels were expressed as concentration in pg/mL in RHE inserts stimulated with the bacteria and treated with the serum in toto compared to positive control (Ctrl+, RHE inserts stimulated with *C. acnes* and treated with DPBS), as shown in Figure 9. The data show a remarkable effect on the release of the IL-8, leading to a statistically significant reduction in the cytokine levels after 24 h of treatment and pro-inflammatory stimulation, compared to stimulated control (Ctrl + *C. acnes*).

## 3. Discussion

Acne is a common juvenile skin inflammatory disorder that was recently investigated also in association with the prolonged use of the mask as personal protective equipment (PPE) during the COVID-19 pandemic [13]. The massive production of sebum by the sebaceous glands (SG), the *C. acnes* hyperproliferation and hyper-keratinization of the pilosebaceous units (PU) are the main processes involved in acne development. For acne treatment, the sebum excretion rate (SER) reduction is one of the most relevant therapeutic targets. Usually, it can be achieved by topical application of products containing functional ingredients as well as oral administration based on retinoids or hormones (estrogens and anti-androgens compounds) [10]. Topical formulations should include keratolytic active ingredients (acids) that unblock sebaceous follicles and prevent their occlusion, anti-seborrheic active ingredients to decrease the SER, humectants and emollients that promote the renewal of skin barrier function, and antimicrobial and anti-inflammatory active compounds to contrast microbial hyper-proliferation. A recent study clarifies that for the treatment of “Maskne”, the common compounds used for acne-prone skin are not recommended. Gentle cleansers with antibacterial active ingredients are proposed for maintaining a healthy skin microbiota, as well as avoiding salicylic acid, alpha hydroxy acids and retinols. To restore a healthy skin barrier function, emollient (lanolin and soy sterols) or occlusive (e.g., petrolatum and mineral oil) active ingredients are not recommended for their potential to induce irritant contact dermatitis under occlusive face mask wear. On the other hand, a valid solution is represented by ceramide/lipid mixtures with anti-inflammatory ingredients [25]. The enriched formulation of the acne stress control serum contains a wide variety of compounds that make it useful for the treatment of acne-prone skin but also for the recent maskne mechanical form. In particular, a key role is played by ceramide’s mixture, Niacinamide, 10-Hydroxydecanoic acid (10-HDA) and Sebacic acid [26,27,28]. 

Starting from careful scientific research related to the functional compounds contained in the formulation, an experimental design to investigate its stability and effectiveness was carried out. Firstly, our study was focused on the biophysical and microbiological stability of the formulation in order to study its rheological behavior but also the quality assurance of its preservative system. Cosmetics could be defined fluids and they are divided in Newtonian, with the constant viscosity value increasing the shear rate, and non-Newtonian, subdivided into dilatant and pseudoplastic fluids. The first one has a directly proportional viscosity and shear rate, while for pseudoplastic fluids, the correlation was inversely proportional [18]. Furthermore, under both conditions evaluated, the serum shows a non-Newtonian shear thinning behavior (pseudoplastic) with a tendency to decrease its viscosity at increasing shear rates. In the packaging, at rest, high viscosity avoids a phase-separation effect, while low viscosity at medium–high shear rate values is an index for a better application and absorption of the product on the human skin [29]. The results showed that the serum presents a storage modulus (G’) increased with respect to the loss modulus (G’’). These features are maintained at 25 °C (condition at rest simulating the storage in the packaging and during extrusion) and at 35 °C (simulating application of the serum in contact with the human skin), demonstrating a solid-like behavior of the serum at either condition. The effectiveness of the preservative system was evaluated according to the reference standard Guideline ISO 11930:2012, and the results obtained comply with the acceptability criteria [20]. Nowadays, for targeting cosmetic applications, in vitro study is a valid approach for the screening assessment of cosmetic final formulation activity. The cosmetic effectiveness has been evaluated by preliminary cytotoxicity investigation in order to set up co-culture colonization models with the most relevant acne-inducing bacterium in monolayer and reconstructed 3D models [6]. The serum effect on cytotoxicity/viability has been investigated through the LIVE/DEAD^®^ assay for mammalian cells, and the images acquired under a fluorescence microscope show a dose-dependent reduction in the ratio between live and dead cells. In fact, higher serum concentrations show an increase in the number of dead cells (in red) compared to untreated cells (Ctrl). Data on cytotoxicity have been confirmed by MTT assay performed also with the purpose to select the first three concentrations with a viability greater than 80% (0.078–0.156–0.313 mg/mL) for the following efficacy test. The ability to modulate epidermal differentiation markers, as cytokines, is a representative index to evaluate the anti-inflammatory activity of a formulation. Therefore, the *C. acnes* bacterial culture has been used to infect monolayer eukaryotic cells (HaCaT), as the mostly represented cell line in the stratum corneum, in the presence and absence (Ctrl+) of the serum. The soothing effect of the serum has been estimated quantifying the IL-1α levels by immunochemistry using an enzyme-linked immunosorbent assay (ELISA). The results show a significative reduction (42–51% and 25%) of the pro-inflammatory cytokine IL-1α at all tested concentrations (0.078, 0.156 and 0.313 mg/mL, respectively) with respect to cells stimulated only with *C. acnes* (Ctrl+) demonstrating good anti-inflammatory activity. After verifying the effectiveness of this experimental model, the same claim has been investigated on a reconstructed human epidermis (RHE) model stimulated in the medium (breached barrier model) with *C. acnes* to mimic a deep inflammation of the SG [30]. A preliminary MTT was necessary to confirm that the 24 h of stimulation and treatment did not interfere with the viability of the reconstructed tissues. Among the pro-inflammatory markers up-regulated during *C. acnes* colonization, IL-8 is more affected by bacterium presence, and its gene expression is significantly increased after 24 h of stimulation [11,30]. The adopted experimental model proved to be very effective for the purpose, including the selected 24 h of treatment and stimulation to mimic the deep absorption of the serum following its application in an inflammatory context similar to the SG status in acne-affected skin. The results show that the conditions adopted do not interfere with the reconstructed tissue viability (≥50% in all conditions tested), and the stimulation with *C. acnes* lead to a significative increase in IL-8 expression (+73%) that was an ideal condition to investigate the soothing effect of the serum showing a significative reduction of IL-8 amount (−55%) respect the positive control. The antimicrobial activity of the serum was evaluated by contact for different interval times (8–24 h) and different serum concentrations (75–50% and following dilution 1:2 until 1.56%) with the reference *C. acnes* strain. The results show an antimicrobial activity of the formulation since by the shortest contact time (8 h) with a bacterial load reduction of 99.9% at the 50% of the serum. The longest contact time of 24 h confirms a sensible reduction of the microbial load already at the tested concentration of 50% with a growth inhibition at a concentration of 25% slightly more evident compared to the 8 h of contact. Since recent studies identify the *C. acnes* biofilm formation as a risk factor in chronic bacterial infections, it was our interest to investigate the potential serum ability to eradicate a preformed biofilm [6,7]. Currently, there is no standard method for assessing new anti-biofilm compounds, but a valid mean for the quantification of bacterial biofilms is the determination of the metabolic activity through MTT assay after treatment of a preformed biofilm [31]. The results show a significant concentration-dependent reduction, demonstrating that after absorption, the serum can be effective against the biofilm-related PU mechanical occlusion. Studies conducted on a 3D skin model are more relevant for cosmetic purposes and in particular RHE and human reconstructed corneal epithelium (HCE) are realistic for the skin and eye environment and ideal for the cosmetics study. In our experimental design, RHE and HCE models were chosen to evaluate the irritating potential of the anti-acne serum. The results show a RHE cell viability of 94% and IL-1α levels below 9 IU/mL (irritation threshold), demonstrating that the serum is not cytotoxic or irritating in a 3D model closer to human skin. The data collected on HCE model show a cell viability of 82.55%, demonstrating that the serum can be classified as No cat. or not belonging to the two risk classifications for eye irritation (Cat. 2) and serious eye damage (Cat. 1), features that make it safe for the application in the periocular zone.

In conclusion, the rheological characterization defines the solid-like and Pseudoplastic behavior of the serum at rest and simulating extrusion and application. This feature, in association with the quality assurance of the preservative system, makes the formulation suitable for topical applications. Afterwards, the bibliographic research and the experimental plan adopted allowed to define the tested product as an effective means for acne-prone skin treatment due to its enriched formulation that presents high biocompatibility, antimicrobial, biofilm eradication and anti-inflammatory activity as well as no potential irritant risk.

## 4. Materials and Methods

### 4.1. International Nomenclature of Cosmetic Ingredients (INCI)

The functional classification of ingredients contained in the topical serum for acne-prone skin (acne stress control serum) provided by Matex Lab Spa (Brindisi, Italy), is summarized in Table 3. The formulation contains a synthetic blend of omega hydroxy acids, sebacic and hydroxy-decanoic acids (10-HAD) that mimics the composition of royal jelly in controlling and reducing excess sebum production [27,28]. Among the main active ingredients, Niacinamide (4%) has anti-inflammatory properties, Ceramides improve barrier function, and Dimethylmethoxy Chormanol provides antioxidant protection to eliminate irritating metabolites caused by acne-related sebum production [26]. Emollients help to soften the external layer of the skin, increasing the ability of the skin to hold water and playing an important role in conferring peculiar rheological properties [32].

### 4.2. Rheological Measurements

To investigate the steady shear flow behaviour of the serum, the shear rate ramp test was performed with rotational rheometer Kinexus Plus (Malvern Panalytical, Worcestershire, UK) at a fixed temperature of 25 °C, but the 100 s^−1^ was also evaluated at 35 °C in order to reproduce the external body temperature during the topical application of the product. This test measures the shear viscosity (η) after an increase in the shear rates and it determines if a material is Newtonian or non-Newtonian. The topical application procedure can be subdivided into steps, so a wide range of shear rate values (0.01–1000 s^−1^ logarithmic scale) was evaluated for a prediction of rest, extrusion and application of the cosmetic [19]. A preliminary amplitude sweep test was performed in the range of 0.1–10% at 25 and 35 °C in order to define a constant strain value at which to perform a frequency sweep test. The third test was conducted in isothermal conditions (25 °C and 35 °C), with a working gap set at 1.0 mm, within the range of 0.1–20 Hz at a shear strain of 0.2%, to remain in the linearity limits of viscoelasticity (determined with the preliminary amplitude sweep test). This test could be used as a criterion to evaluate the stability of the product at rest or subjected to small external stress varying the oscillation frequency. The frequency sweep test is useful to determine the storage modulus (G’) that represents the elastic component, loss modulus (G’’) that is the viscous component, and tangent phase angle (tan δ) that allows to determine which component prevails between G’ and G’’. Tan δ is the result given by the G’’/G’ ratio, so in case G’’/G’ < 1, the elastic component prevails, while if G’’/G’ > 1, the viscous component is higher than the elastic component; on the other hand, in case G’’/G’ = 1, the two parameters are equal [16]. Measurements were made in triplicate, using a plate-plate geometry (PU20 SR2467 SS) with a working gap set at 1.0 mm and a fresh sample loaded for each run. The data processing was performed with rSpace for Kinexus software (Malvern Panalytical, Worcestershire, UK).

### 4.3. Evaluation of Microbiological Stability

The challenge test was performed according to the Method ISO 11930:2012 [20]. The preservative stability of the formulation was screened against the reference strain: *P. aeruginosa* ATCC 9027, *S. aureus* ATCC 6538, *E. coli* ATCC 87394, *C. albicans* ATCC 10231 and *A. brasiliensis* ATCC 16404 provided by Mecconti S.A.R.L. Sp.z.o.o. (Warszawa, Poland). Bacterial strains were incubated for 24–48 h at 35 ± 2 °C on Tryptic Soy Agar (TSA, Condalab, Madrid, Spain), *Candida albicans* was incubated for 48–72 h at 25 ± 2 °C on Sabouraud Dextrose Agar (SDA, Condalab, Madrid, Spain), and *A. brasiliensis* was incubated for 72–120 h at 23 ± 2 °C on Potato Dextrose Agar (PDA, Condalab, Madrid, Spain). EUGON LT 100 Broth (Condalab, Madrid, Spain) was used as a neutralizer to inactivate the preservative system of the serum. Initial inoculi were 1,000,000 for bacteria colony forming unit (CFU)/g, 100,000 and 10,000 CFU/g for *C. albicans* and *A. brasiliensis*, respectively. The test was followed for 28 days (time intervals analyzed: 7–14 and 28 days).

### 4.4. Cell Cultures and Bacterial Strain

The immortalised human keratinocytes cell line (HaCaT, BS code CL 168) was provided by I.Z.L.E.R. (Istituto Zooprofilattico della Lombardia ed Emilia Romagna, Brescia, Italy) and was cultured in a complete medium constituted by High Glucose Dulbecco’s Modified Eagle’s Medium (DMEM, Biowest, Nuaillé, France) supplemented with 10% Fetal Bovine Serum (FBS, Gibco-Fisher Scientific, Waltham, MA, USA), and 1% of L-glutamine (Capricorn Scientific, Ebsdorfergrund, Germany) penicillin (100 U/mL) and streptomycin (100 µg/mL) (Capricorn Scientific, Ebsdorfergrund, Germany), in conditions of complete sterility and maintained in incubation at 37 °C with 5% carbon dioxide (CO_2_) atmosphere. 

The strain *C. acnes* ATCC 11827 (Microbiologics, St Cloud, MN, USA) was cultured in Brain Heart Infusion (BHI, VWR Chemicals, Milano, Italy) broth for the liquid growth of the strain and Sheep Blood Agar (Biolife Italiana, Milan, Italy) for the growth of colonies in plate. The bacteria were cultured in an anaerobic atmosphere using BBL GasPak systems (Becton Dickinson Microbiology Systems, Cockeysville, MA, USA).

Reconstructed human epidermis (RHE) 3D model (Episkin^®^ Laboratories, Lyon, France) was supplied by SkinEthic™ laboratories; it is a reconstructed tissue from normal human keratinocytes grown for 17 days that consists in a fully differentiated epidermis layered on a 0.5 cm^2^ inert polycarbonate filter. 

Human reconstructed corneal epithelium (HCE) tissues and maintenance medium were purchased from Episkin^®^ (Lyon, France). HCE inserts consist of immortalized human corneal epithelial cells stratified and differentiated into a 3D tissue closed to normal human corneal epithelium. The reconstructed architecture contains multi-lamellar layers including columnar basal cells, transitional wing cells and superficial squamous cells [33]. 

Two different media were used for maintenance and growth of both reconstructed models (Episkin^®^ Laboratories, Lyon, France).

### 4.5. Determination of the Antimicrobial Activity against C. acnes 

The product was tested against *C. acnes* to evaluate its ability to reduce the growth after contact for different time intervals (8–24 h). Briefly, the bacterial strain was cultured on sheep blood Agar selective medium for 72 h at 37 °C under anaerobic condition, in order to obtain the separation of the single colony and analyse their morphological uniformity. At the end of the incubation time, the bacterial cells were collected by a sterile loop and inoculated in BHI medium under anaerobic conditions for 24 h. The day before, the antimicrobial activity was evaluated placing in contact in a sterile 24-well plate, the product diluted in BHI broth (range tested 75–50% and following dilutions in a ratio 1:2 until 1.56%) and the bacterial suspension 10^4^ CFU/mL final concentration. Triplicate samples were performed for each test concentration and a range of tested concentrations no-inoculated were used as negative control.

### 4.6. C. acnes Biofilm Eradication Assay

The metabolic activity of the preformed biofilm was determined by the 3-[4,5-dimethylthiazol-2-yl]-2,5 diphenyl tetrazolium bromide (MTT) assay with the same modifications [33]. For the cultivation of biofilm, 200 µL/w of a broth culture (in BHI) at 5 × 10^7^ CFU/mL density were seeded in a flat-bottomed polystyrene 96-well plate (VWR, Milano, Italy) and incubated anaerobically at 37 °C for 24 h under static conditions [21]. After incubation, the biofilm formation was confirmed by microscopic visualization, fresh BHI for negative control, trans-resveratrol, 3,5,4′-trihydroxy-trans-stilbene (Resveratrol, Millipore, Burlington, MA, USA) at 394 µg/mL concentration and treatments (50% and following 1:2 dilutions, range tested 0.078–50%) were added to each well (200 µL/w) and the plate was incubated for an additional 24 h at 37 °C in an anaerobic atmosphere [7,34]. Based on the density and solubility, 50% of the product was selected as the first concentration to not interfere with the formation of the biofilm. The well content was discarded gently using a single-channel micropipette, and each well was washed twice with 200 µL/w of Dulbecco Phosphate Buffer Saline (DPBS, Sigma Aldrich, St. Louis, MO, USA) and air-dried at 60 °C for 60 min to heat-fix the attached bacteria. The MTT (Merck, Darmstadt, Germany) solution (5 mg/mL in DPBS) was pipetted in each well (100 µL/w), and the plate was incubated for 3 h at 37 °C. The insoluble formazan was dissolved in 100 µL/w of Dimethyl Sulfoxide (DMSO, Honeywell, Charlotte, NC, USA) and the optical density (OD) was measured at 570 nm using a microplate rider (MultiSkan, Thermo Scientific, Waltham, MA, USA) in order to calculate the metabolic activity as follows:

Biofilm metabolic activity (%) = [OD_570 nm_ test product/OD_570 nm_ negative control] × 100.

### 4.7. In Vitro HaCaT Cytotoxicity/Viability Assay

Cell viability was evaluated using MTT assay. HaCaT cells were homogeneously seeded in a 96-well plate at 1.5 × 10^4^ density for each well and incubated at 37 °C, with a 5% CO_2_ humidified atmosphere. After 24 h, cells were treated with the serum, starting from 10 mg/mL, following serial dilution (1:2) in cell medium (tested range 0.078–10 mg/mL) supplemented with 0.5% FBS. Untreated cells were used as control (Ctrl). The test was carried out in three replicates for each dilution. After 8 h of treatment, cells were incubated with 1 mg/mL MTT solution at 37 °C for 2 h. After, the medium was discarded from each well and isopropanol (VWR Chemicals BDH, Milan, Italy) was added to dissolve formazan crystals; the absorbance was read at a wavelength of 570 nm. Cell survival was calculated by measuring the difference in OD of the tested product with respect to control (untreated cells). A reduction in cell viability by more than 20% is considered a cytotoxic effect [35].

Cell viability (%) = [OD_570 nm_ test product/OD_570 nm_ negative control] × 100

In order to confirm the dose-dependent cytotoxic effect of the serum, the LIVE/DEAD^®^ viability kit for mammalian cells (Invitrogen, Paisley, UK) was used to stain the live and dead cells after treatment with different serum concentrations. Firstly, HaCaT cells were homogeneously seeded in 22 mm sterile glass coverslips contained in 32 mm Petri dishes, at a 6.5 × 10^5^ density for glass and incubated at 37 °C, with 5% CO_2_ humidified atmosphere. After 24 h, cells were washed with DPBS and treated with the serum at the same concentrations tested in the MTT assay (tested range 0.078–10 mg/mL) supplemented with 0.5% FBS. Untreated cells were used as control (Ctrl). The test was carried out in duplicate for each dilution. After 8 h, treatment was removed, the cells were washed in DPBS and the LIVE/DEAD^®^ cell viability kit was employed to differentiate live cells from the dead cells by double-staining with green-fluorescent 2 μM Calcein-Acetoxymethyl (Calcein AM, Invitrogen, Paisley, UK), which indicates intracellular esterase activity, and red-fluorescent 4 μM Ethidium homodimer-1 (EthD-1, Invitrogen, Paisley, UK), which indicates the loss of plasma membrane integrity. Following incubation, the wet coverslips were inverted and mounted on the microscope slide for the view of the labelled cells under a fluorescence microscope (DM6B Leica Widefield, Wetzlar, Germany) at 20× magnification.

### 4.8. Evaluation of Anti-Inflammatory Activity on HaCaT Cell Line C. acnes Stimulated

In order to use *C. acnes* as pro-inflammatory stimulus on keratinocytes, the strain was cultured in BHI broth for 72 h at 37 °C under anaerobic condition. The log-phase bacterial culture was harvested, washed with DPBS (×3) and incubated at 80 °C for 30 min to kill the bacteria. The heat-killed *C. acnes* was stored at 4 °C until use [36]. HaCaT cells were seeded in a 96-well plate and treated with the anti-acne serum product at the concentrations determined by a preliminary MTT test (0.078–0.0156–0.313 mg/mL). To perform the test, 30 min after the treatment, cells were stimulated with heat-killed *C. acnes* culture (10^7^ CFU/mL). HaCaT cells stimulated and untreated represented the positive control (Ctrl+). 

Therefore, to investigate the potential anti-inflammatory activity of the anti-acne serum in HaCaT cells stimulated with *C. acnes* for 7.5 h, the interleukin (IL)-1α expression was evaluated using an enzyme linked immunosorbent assay (ELISA) kit (Human IL-1α ELISA kit, Thermo Scientific, Waltham, MA, USA). Samples were added to each well in duplicate and the assay was performed according to the manufacturer’s instructions. The absorbance was then read at 450 nm using a microplate reader and the concentration of IL-1α (pg/mL) was calculated by plotting the mean absorbance for each sample on the five parameters logistic (5PL) standard curve representing best fit.

### 4.9. Evaluation of Skin Irritation on 3D Reconstructed Human Epidermis (RHE)

The potential skin irritation of the serum on in vitro RHE was evaluated, according to Method ISO 10993-10: 2010 [22]. After arrival, the RHE inserts were placed in a maintenance medium (6-well plate) under sterile conditions and incubated at 37 °C, 5% CO_2_ overnight. After 24 h, the serum was applied in toto on the surface of the epithelium insert for 42 min at 32 μL/cm^2^ concentration. This contact can mediate the penetration of various compounds to the deeper layers of the epidermis similar to physiological conditions. In parallel, the other inserts have been treated with DPBS and with the positive control, consisting of a 5% (*w*/*v* in water) solution of sodium dodecyl sulphate (SDS, Sigma Aldrich, St. Louis, MO, USA), representing the irritating treatment. 

Each condition, including positive and negative controls, was evaluated in quadruplicate. At the end of the treatment, RHE inserts were rinsed with DPBS (×25) with a continuous flow maintained at 5–8 cm distance, avoiding splashing and contaminations; the bottom of each insert was hit on sterile gauze and transferred into 6-well plates for the incubation at 37 °C, 5% CO_2_, 95% humidified atmosphere for 42 h. Treated inserts were transferred to a 24-well plate filled with 300 µL of MTT solution (1 mg/mL), prepared according to the directions given by the company SkinEthic™, and incubated for 3 h at 37 °C, 5% CO_2_. After extraction with isopropanol, the OD of the samples was quantified by spectrophotometry at a wavelength of 570 nm using a microplate reader. 

The viability of the RHE treated with the serum was calculated as a ratio of the corrected optical densities of the sample over the negative control (untreated sample).

Cell viability values ≤ 50% are index of irritation. 

To better evaluate the skin irritation effect of the cosmetic serum, as well as the viability resulting from the direct contact of the product on the inserts, the levels of IL-1α released after treatment were also measured after 42 h of recovery time by an ELISA kit (Diaclone, Besançon cedex, France) following the manufacturer’s instructions. The absorbance was then measured at 450 nm and the IL-1α quantification was obtained plotting the mean absorbance of each sample with a linear regression standard curve (3.9–250 pg/mL).

### 4.10. Evaluation of Eye Irritation on Human Reconstructed Corneal Epithelium (HCE)

The evaluation of the ocular irritation potential of the serum was assessed with SkinEthic™ HCE model, a reconstructed human cornea-like epithelium. It constitutes a 3D multi-lamellar architecture closer to the human cornea and a relevant model for the study of potential irritating risk of a product applied in periocular zone [33]. Upon receipt, the HCE inserts were placed in maintenance medium (6-well plate) under sterile conditions and incubated at 37 °C, 5% CO_2_ overnight. Following this equilibration period, 30 μL of the serum in toto and 10 μL of DPBS were directly topically onto the tissues for 30 min. Inserts representing the negative and positive controls were treated with 30 μL DPBS and with 30 μL methyl acetate (Sigma Aldrich, St. Louis, MO, USA) added with 10 μL DPBS, respectively. All conditions are evaluated in triplicate.

At the end of the exposure, HCE inserts were rinsed with DPBS (×2) and transferred in 24-well plates for the incubation at 37 °C, 5% CO_2_, 95% humidified atmosphere for 30 min. After this recovery time, treated inserts were transferred to a 24-well plate filled with 300 µL of MTT solution (1 mg/mL), prepared according to the directions given by the company SkinEthic™, and incubated for 3 h at 37 °C, 5% CO_2_.

After isopropanol extraction for 4 h, the OD of the samples was quantified by spectrophotometry at 570 nm wavelength using a microplate reader. 

The viability of the HCE treated with the serum was calculated as a ratio of the corrected optical densities of the sample over the negative control (untreated sample) and cell viability values ≤ 60% are index of serious eye damage (Category 1) and eye irritation (Category 2) [23]. 

### 4.11. Evaluation of Soothing Effect on RHE Stimulated Model

*C. acnes* strain was cultured in BHI broth for 72 h at 37 °C under anaerobic condition. The log-phase bacterial culture was harvested, washed with DPBS (×3) and the same heat-killed protocol applied in the monolayer anti-inflammatory investigation was performed incubating 5 × 10^8^ CFU/mL bacteria culture at 80 °C for 30 min, with subsequent storage at 4 °C until use [36]. After arrival, the RHE inserts were placed in maintenance medium (6-well plate) under sterile conditions and incubated at 37 °C, 5% CO_2_ overnight. After the equilibration time, 100 μL of the bacteria heat-killed culture was added to the RHE medium and the serum was applied in toto on the surface of the epithelium insert for 24 h at 32 μL/cm^2^ concentration. Inserts treated only with DPBS were chosen as a negative control while inserts treated with DPBS but allocated in medium infected with *C. acnes* were used as a positive control. Each condition, including positive and negative controls, was evaluated in duplicate. At the end of exposure, RHE inserts were rinsed with DPBS (×25) and transferred in 6-well plate for the incubation at 37 °C, 5% CO_2_, 95% humidified atmosphere for 42 h. After the recovery time, the viability was evaluated according to the standard MTT protocol described previously [21], and the RHE culture medium was screened for IL-8 pro-inflammatory cytokine by an ELISA kit (Thermo Scientific, Waltham, MA, USA) following the manufacturer’s instructions. The absorbance was then measured at 450 nm using a microplate reader and the IL-8 quantification was obtained plotting the mean absorbance of each sample with a 5PL standard curve (15.6–1000 pg/mL). 

### 4.12. Statistical Analysis

All data are presented as the mean ± standard deviation (SD) or relative standard deviation (RSD%). All graphs and statistical analyses were performed using GraphPad Prism software version 9.0 (GraphPad Software, San Diego, CA, USA). Analysis of variance and significant differences among means were tested by one-way ANOVA, followed by Bonferroni’s multiple comparisons post-test, where appropriate. Differences were considered significant when *p* ≤ 0.05 (*), *p* ≤ 0.01 (**) and *p* ≤ 0.001 (***).

## Figures and Tables

**Table 1:** Shear rate ramp test results obtained with shear rates chosen in order to simulate different conditions of the product at 25 °C and 35 °C [19]. Results are reported as average value ± relative standard deviation percent (RSD%).

Condition of the Product	Temperature (°C)	Shear Rate (s^−1^)	η (Pa s)
Rest	25	10^−2^	495.43 ± 3.26
Extrusion	25	10^3^	0.09 ± 3.97
Application	25	10^2^	0.51 ± 2.78
Application	35	10^2^	0.32 ± 2.18

**Table 2:** Microbial log reduction during the evaluation of the preservative system stability at each time point evaluated (t 7–14—and 28 days) for each strain (*P. aeruginosa* ATCC 9027, *S. aureus* ATCC 6538, *E. coli* ATCC 87394, *C. albicans* ATCC 10231 and *A. brasiliensis* ATCC 16404) investigated in accordance with the reference standard method [20]. NI: no increase with respect to the previous time point analyzed.

Strain	t7 (Days)	t14 (Days)	t28 (Days)	Criteria
*P. aeruginosa* ATCC 9027	≥5 Log	NI	NI	ACCEPTABLE
*S. aureus* ATCC 6538	≥5 Log	NI	NI	ACCEPTABLE
*E. coli* ATCC 87394	≥5 Log	NI	NI	ACCEPTABLE
*C. albicans* ATCC 10231	≥4 Log	NI	NI	ACCEPTABLE
*A. brasiliensis* ATCC 16404	Not required	≥3 Log	NI	ACCEPTABLE

**Table 3:** Functional classification of ingredients contained in the serum used in the study.

Function	Ingredient
Humectant	Propanediol
Smoothing	Niacinamide
Emollient	Cetearyl Alcohol, Butylene, Dimethicone, Cholesterol, Ethylhexylglycerin
Mattifying	Polymethylsilsesquioxane
Emulsifier	Behenyl Alcohol, Stearyl Alcohol, Glyceryl Stearate, Carbomer, Hydrogenated Lecithin, Sodium lauroyl lactylate, Xanthan gum
Solvent	1,2 Hexanediol, 1,10-Decanediol
Skin conditioning	Polyglyceryl-10 Myristate, Phytosterol, Arginine, Caprylic/Capric Triglyceride, 10-Hydroxydecanoic acid, Ceramide 3, Phytosphingosine, Ceramide 6 II, Ceramide 1
Preservative & Perfume	Phenoxyethanol
Chelating Agent	Caprylhydroxamic acid, Disodium EDTA
pH Regulator	Sebacic Acid
Antioxidant	Dimethylmethoxy Chromanol, D-d-tocopherol

## Data Availability

Data are included in the text; raw data are available from the corresponding author.

## Sample Availability

Samples of the analysed serum are not available from the authors.
